# Assessment of Inheritance and Fitness Costs Associated with Field-Evolved Resistance to Cry3Bb1 Maize by Western Corn Rootworm

**DOI:** 10.3390/toxins9050159

**Published:** 2017-05-11

**Authors:** Aubrey R. Paolino, Aaron J. Gassmann

**Affiliations:** Department of Entomology, Iowa State University, Ames, IA 50011, USA; aubreyrpaolino@gmail.com

**Keywords:** *Bacillus thuringiensis*, maize, *Diabrotica virgifera virgifera*, fitness cost, inheritance, resistance management, refuge strategy

## Abstract

The western corn rootworm, *Diabrotica virgifera virgifera* LeConte, is among the most serious insect pests of maize in North America. One strategy used to manage this pest is transgenic maize that produces one or more crystalline (Cry) toxins derived from the bacterium *Bacillus thuringiensis* (Bt). To delay Bt resistance by insect pests, refuges of non-Bt maize are grown in conjunction with Bt maize. Two factors influencing the success of the refuge strategy to delay resistance are the inheritance of resistance and fitness costs, with greater delays in resistance expected when inheritance of resistance is recessive and fitness costs are present. We measured inheritance and fitness costs of resistance for two strains of western corn rootworm with field-evolved resistance to Cry3Bb1 maize. Plant-based and diet-based bioassays revealed that the inheritance of resistance was non-recessive. In a greenhouse experiment, in which larvae were reared on whole maize plants in field soil, no fitness costs of resistance were detected. In a laboratory experiment, in which larvae experienced intraspecific and interspecific competition for food, a fitness cost of delayed larval development was identified, however, no other fitness costs were found. These findings of non-recessive inheritance of resistance and minimal fitness costs, highlight the potential for the rapid evolution of resistance to Cry3Bb1 maize by western corn rootworm, and may help to improve resistance management strategies for this pest.

## 1. Introduction

The western corn rootworm, *Diabrotica virgifera virgifera* LeConte, is a serious pest of maize in the United States [1]. Rootworm larvae feed on the roots of maize, reducing yield and making plants more susceptible to lodging, which can complicate harvest [2,3]. Pruning of one node of roots by larval rootworm feeding is associated with a 17% loss in yield [3]. Management of rootworm has been complicated by the evolution of resistance to several management strategies, including organochloride, organophosphate, carbamate, and pyrethroid insecticides [4,5,6], crop rotation [1,7], and maize that produces insecticidal crystalline (Cry) toxins from *Bacillus thuringiensis* (Bt) [8,9,10,11,12].

Transgenic crops that produce Bt toxins are used in the management of many agricultural pests. Maize producing the Bt toxin Cry3Bb1 was first registered for management of larval rootworm in 2003 [13]. The planting of Bt maize places selection pressure on populations to develop resistance, and laboratory studies have demonstrated the capacity of rootworm populations to evolve Bt resistance quickly [14,15,16]. Populations of western corn rootworm with field-evolved resistance to Bt maize were first identified in 2011 from fields in Iowa with severe root injury to Cry3Bb1 maize that were sampled in 2009 [8]. Other instances of field-evolved resistance to Cry3Bb1 maize, cross-resistance among Cry3Bb1, mCry3A and eCry3.1Ab, and resistance to Cry34/35Ab1 maize have since been identified [8,9,10,11,12,17,18,19,20].

The refuge strategy, in which a portion of the field is planted to a non-Bt host, is one approach to manage the development of resistance to Bt crops. For a maize hybrid with a single Bt trait targeting western corn rootworm, 20% of a field must be planted to non-Bt maize for a spatially segregated refuge (i.e., block refuge) and 10% of the field must be non-Bt maize if the field is a mixture of Bt and non-Bt plants (i.e., blended refuge) [21]. The non-Bt portion of the field, or refuge, serves as a source of susceptible individuals that may mate with resistant insects, thereby producing heterozygous offspring and reducing the number of homozygous, resistant individuals [22]. The delay in resistance to a Bt crop achieved by the refuge strategy may be affected by both the dominance of resistance and fitness costs of resistance [23,24].

Fitness costs occur, in the absence of Bt, when individuals with one or more resistance alleles have lower fitness compared to susceptible individuals [25]. Fitness costs remove resistance alleles from the refuge population, thereby delaying the evolution of resistance [22,24,25,26]. However, both the magnitude and dominance of fitness costs can be altered by ecological factors such as host–plant cultivar or species [27,28,29], the presence of entomopathogens [30,31,32] and competition [33]. To date, fitness costs of resistance have been investigated in rootworm strains with laboratory-selected resistance [34,35,36,37,38] and field-evolved resistance [39], and these studies suggest that fitness costs can vary among strains and experiments. However, fewer data exist concerning the potential for ecological factors to affect fitness cost of Bt resistance for western corn rootworm.

The inheritance of a resistance trait, in particular the functional dominance of resistance, is the degree to which the survival of heterozygous resistant insects on a Bt crop resembles that of homozygous resistant insects [22,40]. At a high dose of Bt toxin, nearly all heterozygous and homozygous susceptible insects are killed by a Bt crop and resistance is functionally recessive [22,24]. A high-dose Bt crop must either produce a concentration of Bt toxin that is 25 times greater than the concentration required to kill a susceptible individual, or kill 99.99% of susceptible individuals [41]. Maize hybrids currently available for management of western corn rootworm do not produce a high dose of Bt toxin [23,42,43], thus, resistance is expected to be inherited as a non-recessive trait.

In this study, we quantified the inheritance and fitness costs of resistance to the Bt toxin Cry3Bb1 in two strains of western corn rootworm with field-evolved resistance to Cry3Bb1 (Monona and Elma). Both strains were collected from fields where the western corn rootworm population imposed a high level of feeding injury to Cry3Bb1 maize, and resistance to Cry3Bb1 maize by western corn rootworm was confirmed with a plant-based bioassay. The Monona strain was collected from field S5 in Gassmann et al. [9], and the Elma strain was collected from field P2 in Gassmann et al. [10]. Heterozygous crosses were established between resistant and susceptible insects to assess inheritance of resistance using a variety of bioassays including single-plant assays, seedling-mat assays and diet-based assays. We also tested for fitness costs of resistance under differing ecological conditions. One experiment, conducted in a greenhouse, tested for fitness costs when larvae were reared on maize plants grown in field soil, and a second experiment, conducted in a growth chamber, examined the effect of competition on fitness costs. The data from these experiments will add to the current knowledge about Bt resistance by western corn rootworm and will aid in improving resistance management for this pest.

## 2. Results

### 2.1. Quantifying Inheritance of Resistance to Cry3Bb1

**Figure 1:**
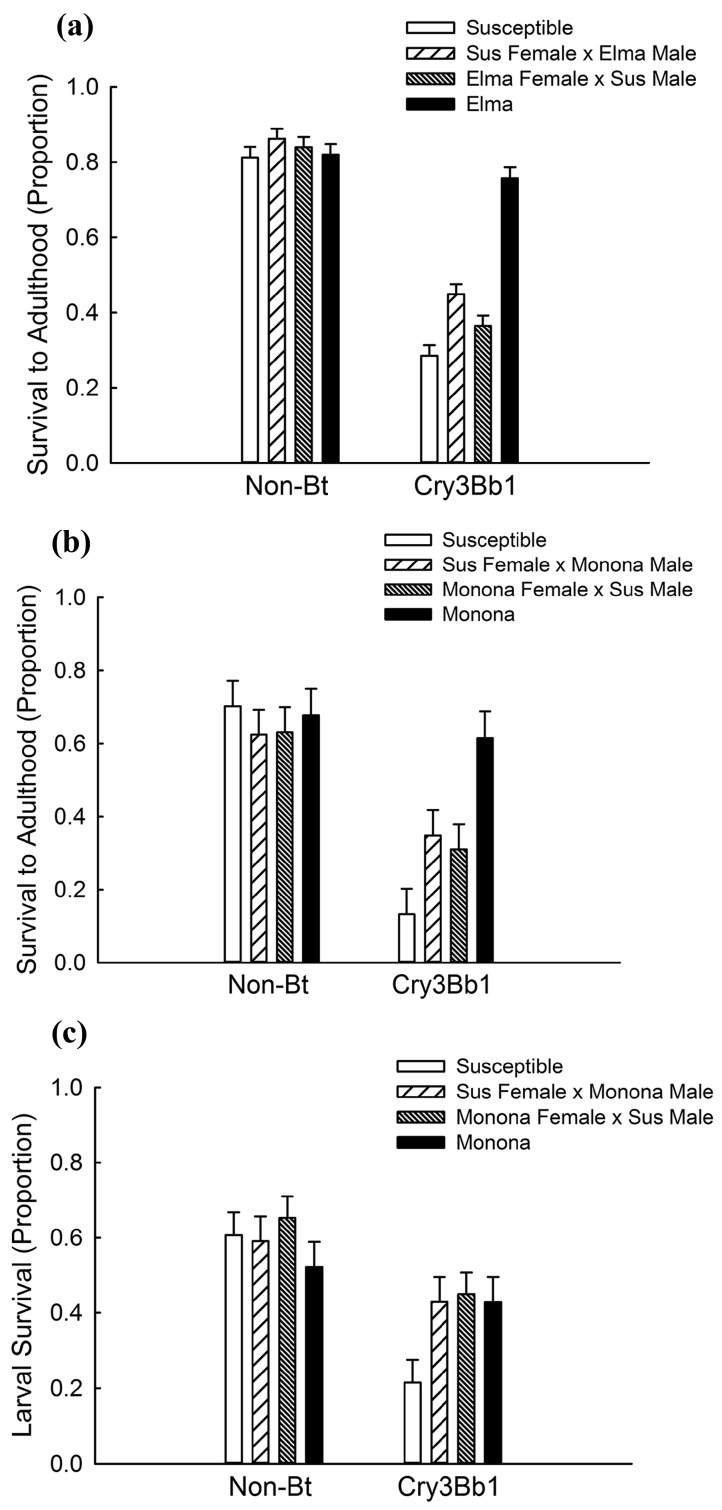
Survival on maize that produces the Cry3Bb1 toxin from *Bacillus thuringiensis* (Bt) and on non-Bt maize for (**a**) seedling-mat bioassay with Elma and Susceptible strains; (**b**) the seedling-mat bioassay with Monona and Susceptible strains; and (**c**) single-plant bioassay with Monona and Susceptible strains. Bar heights represent sample means and error bars are the standard error of the mean.

For the seedling-mat bioassay with Elma, there was a significant interaction between strain and maize hybrid for survival to adulthood when all crosses were included in the model (Elma, Susceptible, Susceptible**♀** × Elma♂ and Elma**♀** × Susceptible♂) (Table 1; Figure 1a). Survival of the heterozygous crosses was similar on non-Bt maize (0.86 ± 0.027 and 0.84 ± 0.028; mean ± Standard Error (SE); linear contrast: df = 1,117; F = 0.37; *p* = 0.5417) but different on Cry3Bb1 maize (0.45 ± 0.027 and 0.36 ± 0.028; linear contrast: df = 1,117; F = 5.06; *p* = 0.0264). Consequently, the two heterozygous crosses were not pooled. The four crosses (resistant, susceptible and the two heterozygous) had equivalent survival on non-Bt maize (all linear contrasts *p* > 0.15) but differed in their survival on Bt maize. Survival on Cry3Bb1 maize was greatest for the Elma strain and there was no difference in survival on non-Bt maize compared to Cry3Bb1 (linear contrast: df = 1,117; F = 2.50; *p* = 0.1167), suggesting complete resistance (Figure 1a). Survival of the Susceptible**♀** × Elma♂ and Elma**♀** × Susceptible♂ was significantly greater than Susceptible on Cry3Bb1 maize (linear contrast: Susceptible vs. Susceptible**♀** × Elma♂: df = 1,117; F = 18.51; *p* < 0.0001; linear contrast: Susceptible vs. Elma**♀** × Susceptible♂ : df = 1,117; F = 4.24; *p* = 0.0416), indicating non-recessive inheritance for both heterozygous crosses. Both heterozygous crosses had lower survival on Cry3Bb1 maize compared to Elma (linear contrast: Elma vs. Susceptible**♀** × Elma♂: df = 1117; F = 62.21; *p* < 0.0001; linear contrast: Elma vs. Elma**♀** × Susceptible♂: df = 1,117; F = 98.48; *p* < 0.0001), indicating that resistance was not dominant. The corrected survival to adulthood on Cry3Bb1 maize was 0.93 (0.76 ÷ 0.82) for Elma, 0.52 (0.45 ÷ 0.86) for Susceptible**♀** × Elma♂, 0.43 (0.36 ÷ 0.84) for Elma**♀** × Susceptible♂, and 0.35 (0.29 ÷ 0.81) for the Susceptible strain. This yielded a resistance ratio for the Elma strain of 2.74 (0.93 ÷ 0.35) and inheritance values of 0.29 for Susceptible**♀** × Elma♂ and 0.14 for Elma**♀** × Susceptible♂, suggesting a paternal component affecting the inheritance of resistance.

For the seedling-mat bioassay with Monona, there was a significant interaction between the rootworm strain and maize hybrid when all crosses were included in the model (Monona, Susceptible, Susceptible**♀** × Monona♂ and Monona**♀** × Susceptible♂) (df = 3,21; F = 8.06; *p* < 0.0001). However, there was no difference in the survival of the two heterozygous crosses on non-Bt (0.65 ± 0.072 and 0.64 ± 0.111; linear contrast: df = 1,21; F = 0.01; *p* = 0.9222) or on Cry3Bb1 maize (0.38 ± 0.062 and 0.35 ± 0.094; linear contrast: df = 1,21; F = 0.25; *p* = 0.6210), and consequently, the heterozygous crosses were pooled. Using the single heterozygous strain, there was a significant interaction between strain and maize hybrid (Table 1; Figure 1b). There was no difference among the three strains on non-Bt maize (all linear contrasts: *p* > 0.15) but the genotypes differed in survival on Cry3Bb1 maize. Monona had the highest survival on Cry3Bb1 maize and survival was equivalent between the Bt and non-Bt hybrids (linear contrast: df = 1,13; F = 0.45; *p* = 0.5163), indicating complete resistance. Survival on Cry3Bb1 maize was lowest for Susceptible and significantly lower compared to survival on non-Bt maize (linear contrast: df = 1,13; F = 50.55; *p* < 0.0001). Survival of heterozygotes on Cry3Bb1 maize was significantly greater than Susceptible (linear contrast: df = 1,13; F = 6.42; *p* = 0.0249), indicating non-recessive inheritance of resistance, but significantly lower than that of Monona (linear contrast: df = 1,13; F = 13.76; *p* = 0.0026) indicating that resistance was not dominant. Corrected survival to adulthood on Cry3Bb1 maize was 0.91 (0.62 ÷ 0.68) for Monona, 0.52 (0.33 ÷ 0.63) for the heterozygous crosses, and 0.20 (0.14 ÷ 0.70) for Susceptible. The resistance ratio for Monona was 4.55 (0.91 ÷ 0.20) and the inheritance of resistance was 0.45.

With the single-plant bioassay using Monona, we found evidence of a potential interaction between strain and maize hybrid when all crosses were included in the model (Monona, Susceptible, Susceptible**♀** × Monona♂ and Monona**♀** × Susceptible♂) (df = 3,26; F = 2.78; *p* = 0.0610). However, because there was no difference in larval survival between the two heterozygous crosses on non-Bt (0.56 ± 0.050 and 0.63 ± 0.068; linear contrast: df = 1,26; F = 0.62; *p* = 0.4391) or on Cry3Bb1 maize (0.41 ± 0.076 and 0.44 ± 0.065; linear contrast: df = 1,26; F = 0.04; *p* = 0.8370); these data were combined. There was a significant interaction between strain and hybrid when the heterozygous crosses were combined (Table 1; Figure 1c) with equivalent survival of the strains on non-Bt maize (all linear contrasts: *p* > 0.15). Survival of Monona on Cry3Bb1 maize was not significantly different compared to survival on non-Bt maize (linear contrast: df = 1,135; F = 1.30; *p* = 0.2571), indicating complete resistance. On Cry3Bb1 maize, survival was significantly greater for heterozygotes compared to Susceptible (linear contrast: df = 1,135; F = 12.52; *p* = 0.0006), indicating non-recessive inheritance of resistance. Survival of heterozygotes on Cry3Bb1 maize was not significantly different compared to Monona (linear contrast: df = 1,135; F = 0.02; *p* = 0.8930), suggesting that resistance was dominant on single plants. Corrected larval survival was 0.83 (0.43 ÷ 0.52) for Monona, 0.70 (0.44 ÷ 0.63) for heterozygotes, and 0.34 (0.21 ÷ 0.61) for Susceptible. This produced a resistance ratio for Monona of 2.44 (0.83 ÷ 0.34) and inheritance of 0.73.

**Figure 2:**
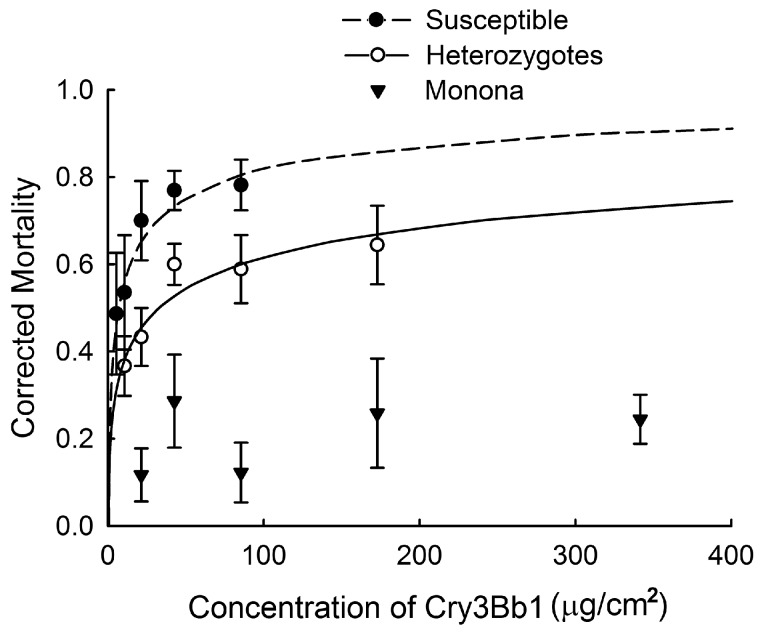
Larval mortality in diet-based bioassays for Susceptible and Monona strains, and heterozygotes. Data were adjusted for control mortality with Abbott’s correction. Points represent sample means, error bars are the standard error of the mean, and lines are probit analyses.

In the diet-based assay with Monona (Table 2; Figure 2), data from the heterozygous crosses were pooled. We were able to calculate the lethal concentration that killed 50% of the population (LC_50_) and 95% fiducial limits for the Susceptible strain and heterozygous cross. In the case of Monona, mortality never exceeded 50%, even at the highest concentration tested (341.60 µg Cry3Bb1/cm^2^). There was a significant difference, as evidenced by non-overlapping 95% fiducial limits, between the LC_50_ values for the Susceptible strain versus the heterozygous crosses (Table 2), indicating non-recessive inheritance of resistance.

### 2.2. Greenhouse Assessment of Fitness Costs

**Figure 3:**
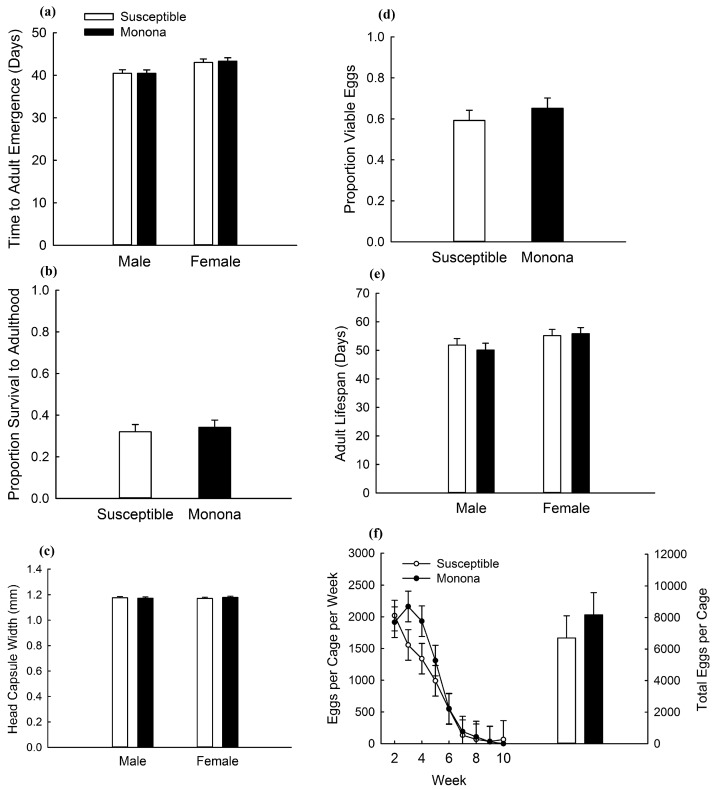
Comparisons of life-history traits for Susceptible and Monona strains on non-Bt maize in an experiment testing for fitness costs. Bar heights represent sample means and error bars are the standard error of the mean. Data are presented for (**a**) developmental rate; (**b**) proportion survival to adulthood; (**c**) adult size; (**d**) egg viability; (**e**) adult lifespan; and (**f**) fecundity.

In the experiment with Monona and Susceptible, strain and its interaction with sex were not significant for any of the variables measured (Table 3 and Table 4; Figure 3). This suggests an absence of fitness costs of Cry3Bb1 resistance in Monona. There was a significant effect of sex on developmental rate with males emerging 2.71 days before females (Table 3; Figure 3a). There was also a significant effect of week on fecundity with egg production decreasing with time (Table 4; Figure 3f).

### 2.3. Effect of Competition on Fitness Costs

**Figure 4:**
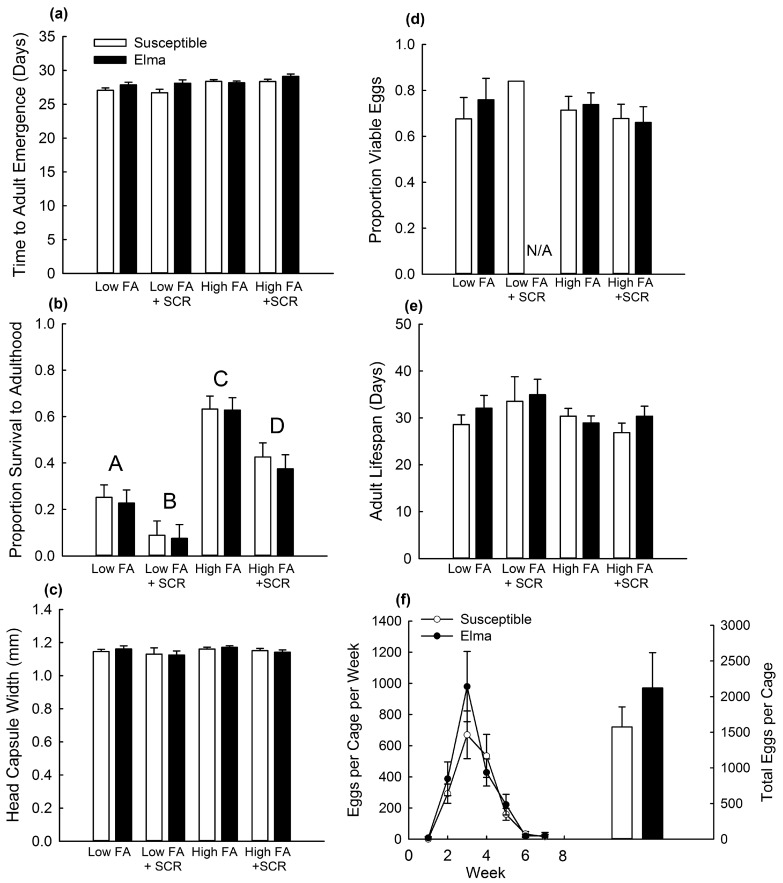
Comparisons of life-history traits for Susceptible and Elma strains on non-Bt maize in an experiment testing for fitness costs. Data are shown for Elma and Susceptible with high or low larval food availability (FA) and in the presence or absence of competition from the southern corn rootworm (SCR). Bar heights represent sample means and error bars are the standard error of the mean. Data are presented for (**a**) developmental rate; (**b**) proportion survival to adulthood; (**c**) adult size; (**d**) egg viability; (**e**) adult lifespan; and (**f**) fecundity. For (**b**) proportion survival to adulthood, letter indicate pairwise differences among means for combinations of food availability and presence or absence of SCR. For low FA + SCR in the graph of (**d**) egg viability; N/A indicates that data on egg viability for Elma were not applicable because no eggs were obtained; additionally, only one observation was obtained for the Susceptible strain, which prevented the calculation of a standard error of the mean.

Survival to adulthood was affected significantly both by food availability and presence of the southern corn rootworm (SCR), indicating an effect of competition on survival (Table 5). Proportion survival to adulthood decreased significantly with either lower food availability or the presence of SCR (Figure 4b). However, survival was not significantly affected by strain or the interaction of strain with other factors, indicating that a fitness cost affecting survival was not present (Table 5; Figure 4b). There was a significant effect of strain on developmental rate (Table 5; Figure 4a). Adult corn rootworm from Elma emerged after 28.07 days ± 0.10 (mean ± SE) while those from Susceptible emerged after 27.76 d ± 0.11, indicating a fitness cost of resistance (Figure 4a). Neither strain nor any interaction with strain were significant for size, adult lifespan, fecundity or egg viability, indicating that no fitness costs were associated with these life-history components (Table 4 and Table 5; Figure 4). For fecundity, there was a significant effect of week and an interaction between week and food availability (Table 4; Figure 4f). Initially, egg production was greater for insects from larval rearing containers with higher food availability (week 3: low food availability = 336 ± 160 eggs per cage; high food availability = 1100 ± 118; mean ± SE) but this difference decreased over time (week 5: low food availability = 66 ± 31; high food availability = 257 ± 51).

## 3. Discussion

Our study investigated the inheritance of resistance and associated fitness costs for two strains of western corn rootworm with field-evolved resistance to Cry3Bb1 maize. For these strains, resistance was non-recessive and minimal fitness costs were detected. Past studies also have documented non-recessive inheritance of Bt resistance for western corn rootworm [16,36,39] while the presence of fitness costs varied among strains and experiments [35,36,37,39]. These findings, and those of other studies, suggest that field-evolved resistance to Cry3Bb1 maize by western corn rootworm was likely facilitated by non-recessive inheritance of resistance traits and similar fitness between resistant and susceptible insects in refuges [42].

Fitness costs of Bt resistance function to remove resistance alleles from a population in the absence of Bt toxins (i.e., within a non-Bt refuge). Resistance alleles accumulate in the refuge population because of selection within Bt fields and subsequent dispersal into refuge populations [44]. When fitness costs are present, selection against resistance alleles in the refuge can delay or reverse the evolution of resistance in a population, compared to when fitness costs are absent [45]. In this study, no fitness costs were detected for the Monona strain in a greenhouse experiment (Figure 3), and only minimal fitness costs were detected for Elma (Figure 4), with increased developmental rate from larva to adult for Elma compared to Susceptible (Figure 4a). Ingber and Gassmann [39] also identified a fitness cost of delayed larval development for a different strain of western corn rootworm (Cresco) with field-evolved Cry3Bb1 resistance. Conversely, Oswald et al. [35] identified no fitness costs of resistance to Cry3Bb1 in laboratory-selected western corn rootworm strains with resistance to Cry3Bb1 maize and found that resistant lines had an increased rate of larval development compared to unselected strains. Results with other strains of Cry3Bb1-resistant western corn rootworm have ranged from finding no fitness costs associated with resistance [36,37,38,39] to costs affecting survival and fecundity [34,39]. Additionally, in this study, there were no effects of competition on fitness costs. Past research has found that the presence of entomopathogens and variation in maize hybrid did not alter fitness costs of Bt resistance in western corn rootworm [37,38]. Although fitness costs of Bt resistance in some pest species can be affected by ecological factors, such effects may be rare for fitness costs of Bt resistance by western corn rootworm [25]. However, future research on other field-relevant variables affecting fitness of western corn rootworm may be usefully in better characterizing potential fitness costs of Bt resistance. Such factors might include overwintering temperature, and the duration of time between larval hatch and establishment of larvae on maize roots [46].

Compared to western corn rootworm, more research on fitness costs of Bt resistance has been conducted on lepidopteran pests, especially the diamondback moth (*Plutella xylostella* Linnaeus) and the pink bollworm (*Pectinophora gossypiella* Saunders), and fitness costs have been observed for both of these species [25]. Carrière et al. [47] found that, on average, survival of Cry1Ac-resistant pink bollworm on non-Bt cotton was 51.5% lower compared to susceptible strains. Likewise, a fitness cost was associated with resistance to Cry1Ac in the diamondback moth [48]. A review of studies by Gassmann et al. [25] found that fitness costs were detected in 34% of experiments that compared life-history traits between resistant and susceptible strains, and in 62% of experiments that tested for a decline in resistance over multiple generations without selection on Bt. Future studies that test for a decline in resistance over time in strains of western corn rootworm with field-evolved resistance should be conducted to better understand the effect that fitness costs might have on resistance to Bt maize by this pest in the field.

Past research has found a positive relationship between the rate of resistance evolution and the effective dominance of resistance (i.e., the degree to which survival of heterozygous individuals on Bt plants resembles that of homozygous resistant insects) [22,40]. We found non-recessive inheritance of resistance for both the Elma and Monona with our plant-based assays (Figure 1). Other studies also have found non-recessive inheritance in Cry3Bb1-resistant strains [16,36,39], suggesting that non-recessive inheritance of resistance to Cry3Bb1 maize is common for the western corn rootworm. In diet-based bioassays conducted as part of this study, the LC_50_ of heterozygous strains was significantly greater than that of the Susceptible strain (Table 2), again suggesting non-recessive inheritance of resistance. The relationship between dominance and dose is expected to influence the effective dominance of Cry3Bb1 resistance for western corn rootworm. When insects are not exposed to a high dose of toxin, as is the case with Cry3Bb1 maize and western corn rootworm, the effective dominance of resistance increases, and resistance is expected to evolve more quickly [22,40,49].

Evidence from field-evolved resistance in other insect species supports the supposition that resistance evolves more quickly when a Bt crop does not produce a high dose of toxin against its target pest, and consequently, resistance is non-recessive [24]. For example, the corn earworm (*Helicoverpa zea* Boddie) has non-recessive inheritance of resistance to Cry1Ac cotton and evolved resistance to Cry1Ac cotton; while the closely-related tobacco budworm (*Heliothis virescens* Fabricius), against which Cry1Ac cotton does produce a high dose of toxin, has remained susceptible [40,50]. In addition to the western corn rootworm and corn earworm, both the maize stalk borer (*Busseola fusca* Fuller) and fall armyworm (*Spodoptera frugiperda* Smith) developed resistance to Bt maize that failed to produce a high dose of toxin against these target pests [24,51,52,53,54].

There were differences in the magnitude of resistance and inheritance of resistance between the two strains, with Elma having a resistance ratio of 2.74 and Monona having a resistance ratio of 4.55 in the seedling-mat bioassays. This, along with differing resistance ratios in other western corn rootworm strains with field-evolved resistance [39], may be the result of differences in the intensity of selection each strain experienced in either the field or the laboratory, or differences in the mechanisms of resistance among strains. Additionally, we found evidence of sex linkage, through a paternal effect, for resistance in Elma but not Monona. Past work also has found evidence of a paternal effect for Cry3Bb1 resistance [36]. However, there was a clear autosomal component of Cry3Bb1 resistance in Elma, with both heterozygous crosses showing increased survival on Cry3Bb1 maize compared to the Susceptible.

There also were differences in the inheritance of resistance between the plant-based bioassays evaluated in this study. While Monona showed non-recessive inheritance of resistance in the two plant-based bioassays (i.e., single plant and seedling mat), heterozygotes in the seedling-mat bioassay had significantly lower survival on Cry3Bb1 maize than Monona (*h* = 0.45; Figure 1b), but by contrast, there was no difference in survival on Cry3Bb1 maize between heterozygotes and Monona in single-plant bioassays (*h* = 0.75; Figure 1c). This difference may be related to the dose of Cry3Bb1 insects experienced in these two assay types, with a higher dose achieved in the seedling-mat assay than the single-plant assay. This hypothesis is supported by higher corrected survival of the Susceptible strain on Bt maize in the single-plant assay (0.34) compared to the seedling-mat assay (0.20). This difference also may have arisen because the seedling-mat assay measured survival to adulthood and the single-plant assay measured larval survival. If additional mortality of the heterozygotes on Cry3Bb1 maize occurred at the end of the larval stadium or during pupation, this would not have been captured by the single-plant assay. In general, the proportion of survival for susceptible insects on V5 to V6 Bt maize plants (i.e., plants at the five to six leaf stage) is 0.00 to 0.04 [10], which is lower than the survival on V6 to V8 maize plants (i.e., plants at the six to eight leaf stage) used in this study (i.e., 0.34). However, due to low survival of the non-diapausing strains studied here on V5 to V6 plants, it was not possible to use the same plant-based assay that has been applied to evaluate field populations in other studies (i.e., [8,9,10,11,12,17,20]).

Our findings of non-recessive inheritance and a lack of major fitness costs in western corn rootworm strains with field-derived resistance to Cry3Bb1 suggest that the refuge strategy alone is likely insufficient to delay resistance development. This highlights the need for more diversified management of western corn rootworm through an integrated pest management approach including rotation among management strategies [42]. The use of diverse approaches such as pyramiding of multiple Bt toxins, use of soil-applied insecticide with maize lacking rootworm-active Bt toxins, and crop rotation may help to delay the evolution of resistance to current and future Bt traits for management of western corn rootworm.

## 4. Methods

### 4.1. Rootworm Strains

In total, three strains of western corn rootworm and one strain of southern corn rootworm (*Diabrotica undecimpunctata howardi* Barber) were studied in these experiments. The Susceptible strain of western corn rootworm is a non-diapausing strain that was brought into laboratory culture in the 1970s and never exposed to Bt maize [55,56]. Insects were acquired from the United States Department of Agriculture, Agricultural Research Service, North Central Agricultural Research Laboratory (Brookings, South Dakota) to establish the Susceptible strain at Iowa State University in October 2009 (F_1_). This research used F_28_ to F_35_ of Susceptible.

The Monona and Elma strains are non-diapausing strains of western corn rootworm with field-evolved resistance to Cry3Bb1 maize. Both strains originated from fields where a high level of feeding injury to Cry3Bb1 maize by western corn rootworm was observed, and resistance was confirmed with a plant-based bioassay. In August 2011, adult male western corn rootworms were collected from field S5 in Gassmann et al. [9] to establish Monona and field P2 in Gassmann et al. [10] to establish Elma. Two hundred field-collected adult males were collected to initiate Monona and 142 field-collected adult males were collected to initiate Elma. To generate each strain, field-collected males were crossed with 150 virgin females from Susceptible. Monona was subsequently selected on Cry3Bb1 maize after being backcrossed with the Susceptible strain at a 1:1 ratio twice (F_6_ and F_8_) and selected on Cry3Bb1 maize without backcrossing four more times (F_10_, F_11_, F_14_, and F_15_). Elma was selected on Cry3Bb1 maize after being backcrossed with Susceptible at a 1:1 ratio twice (F_4_ and F_7_) and selected on Cry3Bb1 maize without backcrossing twice (F_10_ and F_11_). In all other generations, the strains were reared on non-Bt maize (Pioneer 34M94, DuPont Pioneer, Johnston, IA, USA). Experiments used F_20_ to F_26_ of Monona and F_18_ to F_20_ of Elma. The adult population size was maintained at ca. 2500 adults for the three western corn rootworm strains, and none of the maize seed used to rear these strains contained any type of pesticidal seed treatment.

In one fitness cost experiment, southern corn rootworm (SCR) was used in addition to western corn rootworm. This SCR strain was generated in October 2013 from 381 SCR adults that were collected from the Sustainable Agriculture Garden at Iowa State University (Ames, IA, USA). All generations were reared on non-Bt maize and maintained at a population size of ca. 900 adults.

### 4.2. Strain Rearing

Adult insects were kept in cages (18 × 18 × 18 cm, MegaView Science Co. Ltd., Taichung, Taiwan) in an incubator (Percival Scientific, Perry, IA, USA) at 25 °C with a 16:8 [L:D] h photoperiod. Adult insects were fed a complete adult diet (western corn rootworm adult diet, product # F9768B-M, Bio-Serv, Frenchtown, NJ, USA) and maize leaf tissue, with a 1.5% agar solid provided as a source of water. A petri dish (150 mm in diameter) of moistened sieved field soil (<180 µm) was used as an oviposition substrate and was replaced two times per week. Larvae were reared on mats of maize seedlings following the methods of Jackson [57] and Ingber and Gassmann [39]. Adult insects were collected from seedling mats and placed into cages.

### 4.3. Quantifying Inheritance of Resistance to Cry3Bb1

Reciprocal crosses were established separately, but in an identical manner between Elma and Susceptible, and between Monona and Susceptible, following Petzold–Maxwell et al. [36]. First, all adults were collected and discarded from seedling mats to remove any adults that may have mated, then virgin adults were collected every 2–3 h to ensure that the adults had not mated. Adults were held separately in Petri dishes and sex of each insect was determined following Hammack and French [58]. Virgin adults were then placed in one of four cages: Susceptible**♀** × Susceptible♂, Susceptible**♀** × Resistant♂, Resistant**♀** × Susceptible♂, and Resistant**♀** × Resistant♂. Crosses between Susceptible and Elma were established between 18 July and 26 September 2014 using F_28_ and F_29_ of Susceptible and F_18_ and F_19_ of Elma, with cages maintained at an average population size of 109 ± 34 adults (mean ± SD). Crosses between Susceptible and Monona were established between 10 December 2014 and 9 October 2015 using F_31_ to F_35_ of Susceptible and F_20_ to F_26_ of Monona, and maintained at an average population size of 133 ± 41 adults.

*Seedling-Mat Bioassay.* The seedling mat bioassays were conducted between 21 August and 21 November 2014 using the Susceptible and Elma crosses and between 21 February and 9 December 2015 using the Susceptible and Monona crosses. Assays followed Ingber and Gassmann [39]. Briefly, seedling mats of either Cry3Bb1 maize (DCK 62-63, Monsanto Co., St. Louis, MO, USA) or the non-Bt near isoline (DCK 62-61) were grown in 0.5-L plastic containers (RD-16 Placon Corporation, Madison, WI, USA) for 7 days in an incubator (25 °C; photoperiod 16:8 [L:D] h), after which time 25 neonate larvae (<24 h old) from one of the four crosses (i.e., Susceptible**♀** × Susceptible♂, Susceptible**♀** × Resistant♂, Resistant**♀** × Susceptible♂, or Resistant**♀** × Resistant♂) were placed on the maize root tissue. After 1 week, the seedling mat, soil and larvae from a 0.5-L container was transferred to larger maize seedling mat held in a 1-L plastic tray (C32DE; Dart Container Corporation, Mason, MI, USA) and this larger seedling mat always used the same maize hybrid that was used in the corresponding 0.5-L container. After 1 week, trays were checked for adult emergence three times per week and this continued until no adults were collected from a replicate for 14 days. A replicate consisted of a one non-Bt seedling mat and one Cry3Bb1 seedling mat for each of the four crosses. The experiment with Elma and Susceptible consisted of nine blocks with two replicates per block and the experiment with Monona and Susceptible consisted of 12 blocks with two replicates per block.

*Single-Plant Bioassay.* This experiment was conducted from 29 January to 11 November 2015 and used crosses between Susceptible and Monona. An initial single-plant bioassay was conducted following the methods of Gassmann et al. [10], but due to low larval recovery for these non-diapausing strains, the assay was modified to use older maize plants with more root tissue, to more closely resemble the seedling mats on which these non-diapausing strains were reared. The experiment consisted of 12 blocks with each block containing two non-Bt maize plants and two Cry3Bb1 maize plants for each of the four crosses. Maize plants were grown in a greenhouse and held singly in 1-L plastic containers (Product #22373; Placon Corporation, Madison, WI, USA) filled with 750 mL of potting medium, following Gassmann et al. [8]. Containers received 300 mL water just before seeds were planted (depth = 5 cm). Plants were watered as needed and received 100 mL of fertilizer solution, weekly, beginning 2 weeks after planting (4 mg/mL Peters Excel 15-5-15 Cal-Mag Special; Everris NA Inc., Dublin, OH, USA). When plants had reached V6 to V8, they were trimmed to a height of 20 cm and 12 neonate larvae (<24 h old) were placed on the base of each plant. Containers were placed in an incubator (24 °C, 65% RH, 16/8 L/D) and watered as needed. After 14 d, the aboveground plant material was removed and contents of the container (soil, roots and larvae) were placed on a Berlese funnel for 4 days to extract larvae.

*Diet-Based Bioassay*. Diet-based bioassays were conducted between 14 March and 28 November 2015 and followed Siegfried et al. [59]. Diet-based bioassays used Susceptible and Monona strains, and their reciprocal crosses. Eggs were incubated in soil (26.7 °C, 67% RH, 0/24 h L/D) until hatching began. Soil was then washed from the eggs, and any remaining debris separated by salt flotation [60]. Eggs were surface sterilized in a 2% bleach solution followed by a 0.085% Roccal-D Plus solution (Pfizer, Inc. New York, NY, USA), and placed on a moistened coffee filter atop a 1.8% agar solid held in a 0.5-L container. Monsanto Corporation (St. Louis, MO, USA) provided 96-well plates with diet, a solution of Cry3Bb1 toxin, and buffer [39,59]. Toxin, dissolved in buffer, was overlaid on the diet at six concentrations, which varied by cross due to anticipated differences in susceptibility to Cry3Bb1. The concentrations tested were: Susceptible = 85.40, 42.70, 21.40, 10.70, 5.40, µg Cry3Bb1/cm^2^, and a control with only buffer and no toxin; heterozygote = 170.80, 85.40, 42.70, 21.40, 10.70 µg/cm^2^, and a control; Monona = 341.60, 170.80, 85.40, 42.70, 21.40 µg/cm^2^, and a control. Each bioassay plate consisted of 12 larvae per concentration, with a total of 72 larvae per plate. One neonate larva was placed in each well. The plate was then covered with clear plastic adhesive and held in an incubator for 5 days (26.7°C; 67% RH, 0/24 L/D). After 5 d, the plates were checked for survival (defined as showing movement when prodded). For each plate, survival of at least eight of 12 larvae in control wells was used as the threshold for a successful plate. Six of 12 plates were successful for the Susceptible strain, four of 12 plates were successful for Susceptible ♀ × Monona ♂, five of 13 plates were successful for Monona ♀ × Susceptible ♂, and five of 16 plates were successful for the Monona strain.

### 4.4. Greenhouse Experiment Testing for Fitness Costs

This experiment used the F_21_ of Monona and F_32_ of Susceptible and occurred from 2 January to 12 June 2015. Non-Bt maize (Mycogen 2K591; Dow AgroSciences, Indianapolis, IN, USA), with seed treatment removed following Gassmann et al. [8], was grown in a greenhouse to the V5–V8 stage, at which time 25 neonate larvae (<24 h old) were placed on the roots of each plant. Pots were covered with chiffon fabric secured around the outside of the pot with rubber bands and tied around the stalk with a twist tie. A replicate consisted of one plant with larvae, and 16 replicates each were established for the Monona and Susceptible.

Adult insects were collected three times per week beginning 3 weeks after larvae were added to pots and this continued until there were 6 consecutive days without emergence. After determining the sex of each insect, adults and placed into cages, with one cage for all individuals that emerged from the same pot. Non-Bt maize plants (08T91CMV, Blue River Hybrids, Ames, IA, USA) also were grown in the greenhouse to serve as a food source for adult rootworm. Cages received chopped maize ear, silk, and leaves from these plants as well as 1.5% agar solid as a source of water, and both were changed three times per week. Each cage contained a petri dish with moistened sieved field soil for oviposition, which was changed once per week. Cages were checked three times per week for dead adults, which were removed and stored in 85% ethanol. Later, sex of adult beetles was determined and their head capsules measured according to the methods of Ingber and Gassmann [39]. Egg viability was quantified at 2, 4, and 6 weeks after a cage was established by placing 25 eggs on a 1.5% agar solid and checking for hatch 5 days per week until there were no newly hatched larvae on 3 consecutive days.

### 4.5. Competition Experiment Testing for Fitness Costs

This experiment was conducted between 12 August and 26 November, 2014 and used F_19_ and F_20_ of Elma, F_29_ and F_30_ of Susceptible, and F_6_ and F_7_ of SCR. The experiment was a fully crossed design with three factors: food availability, presence or absence of SCR as a competing species, and strain of western corn rootworm (Susceptible or Elma). Seedling mats were prepared in 0.5-L plastic containers with either low or high food availability achieved by adding either five or 10 kernels of non-Bt maize (Pioneer 34M94), 60 mL of deionized water, and 200 mL of a 50% field-collected soil and 50% potting soil mixture. After 1 week, seedling mats received 25 neonate larvae of either Elma or Standard. At that time, half of the seedling mats also received 25 neonate SCR larvae. After 7 d, small seedling mats were transferred to larger seedling mats that consisted of either 10 or 20 kernels per tray of non-Bt maize (corresponding to low or high food availability, respectively), 60 mL of DI water, and 500 mL of soil, all of which was placed in a 1-L plastic tray. Larger seedling mats were allowed to grow for 7 days before smaller seedling mats were transferred. Adult western corn rootworm were collected and separated into cages following the same methods as the greenhouse experiment. For each combination of strain × food availability × presence or absence of SCR, there was one replication per block and a total of 10 blocks.

Data were collected and adults maintained as in the greenhouse experiment, with the exception of how food was provided, and that insects were fed a complete adult diet instead of maize ear. In this experiment, we simulated the reduced food availability that adult rootworm experience as maize matures in the field. For 4 weeks, each cage received adult rootworm diet, non-Bt maize leaf, and a 1.5% agar solid, which was changed three times per week. Then, for the next 2 weeks, agar and maize leaf were always present but adult diet was only provided for 1 day per week. After that, cages received only agar and maize leaf.

### 4.6. Data Analysis

All data were analyzed with SAS 9.3 (SAS Institute Inc., Cary, NC, USA). For the seedling-mat and single-plant bioassays, data were analyzed with a mixed-model analysis of variance (ANOVA) (PROC MIXED). Fixed effects were strain, hybrid, and the interaction of strain and hybrid. Random effects were block and all interactions with fixed effects. The significance of random effects was tested with a log-likelihood statistic (-2 RES Log Likelihood) based on a one-tailed χ^2^ test with one degree of freedom [61]. A random effect was included in the model if it was significant at *p* < 0.25 or if higher order interactions including the effect were significant [62]. Pairwise comparisons were first made between the two heterozygous crosses using the CONTRAST statement with a *p*-value of 0.05 to determine if the strains could be combined, and if so, subsequent comparisons were made among a resistant strain, heterozygotes and Standard to characterize the inheritance of resistance.

For the seedling-mat and single-plant bioassays, corrected survival on Cry3Bb1 maize was calculated as the complement of corrected mortality based on Abbott [63]. Resistance ratios were the quotient of corrected survival on Cry3B1 maize for a resistant strain divided by Susceptible. Dominance of resistance (*h*) was calculated based on phenotype using corrected survival on Cry3Bb1 maize with the equation: *h* = (heterozygote − susceptible)/(resistant − susceptible), where 0 = recessive, 1 = dominant, and 0.5 = additive inheritance [64].

For the diet-based bioassay, corrected larval mortality for each plate was calculated based on Abbott [63]. Data were analyzed with a probit analysis to determine LC_50_ values, 95% fiducial limits, and goodness-of-fit based on Pearson χ^2^ (PROC PROBIT).

For the fitness costs experiment with plants grown in the greenhouse, data on proportion survival to adulthood and egg viability were compared between strains with a model I ANOVA (PROC GLM). In the analysis of development rate, head capsule width, and adult lifespan, a mixed-model ANOVA (PROC MIXED) was used. Fixed effects were strain, sex, and their interaction, and the random effect was cage × strain × sex. Fecundity (i.e., egg production) was analyzed with repeated measures ANOVA based on a split-plot design (PROC MIXED) [62]. Fixed effects were strain, week and week × strain and the random effects were cage nested within strain, and week × cage nested within strain. Data on fecundity were transformed by the square root function to improve normality of the residuals.

For the experiment measuring the effect of competition on fitness costs, data were analyzed with a mixed-model ANOVA. The analysis of data on proportion survival to adulthood and egg viability used the fixed effects of strain, food availability (high vs. low), SCR (present vs. absent), and all interactions. The random effects in these models were block and the interaction of block × strain × food availability × SCR. For the analysis of development rate, head capsule width, and adult lifespan, the fixed effects were strain, food availability, SCR, sex, and all interactions. The random effects in these models were block and the interaction of block × strain × food availability × SCR × sex. Egg production was analyzed with repeated-measures ANOVA based on a split-plot design, with the fixed effects of strain, week, SCR, food availability and all interactions among these factors. Random effects were cage (strain × kernels × SCR × block) and week × cage (strain × kernels × SCR presence × block), which are the mean square error terms for this repeated-measures model based on a split-plot design [62]. Data on egg production were transformed by the square root function to improve normality of the residuals. When significant interactions were present among fixed effects, means were compared with a Tukey–Kramer test (TUKEY statement in PROC MIXED) to understand the nature of the interaction.

## Figures and Tables

**Table 1:** Analyses of variance for survival in plant-based bioassays.

Experiment	Effect	df	F	*p*
Elma seedling mat ^a^	Strain	3,117	26.86	<0.0001
	Hybrid	1,117	366.08	<0.0001
	Strain × Hybrid	3,117	27.92	<0.0001
Monona seedling mat ^b^	Strain	2,13	6.98	0.0087
	Hybrid	1,11	41.05	<0.0001
	Strain × Hybrid	2,13	10.32	0.0021
Monona single plant ^c^	Strain	2,135	3.72	0.0268
	Hybrid	1,135	30.14	<0.0001
	Strain × Hybrid	2,135	4.11	0.0185

^a^ Random effect in the model was block (df = 1, χ^2^ = 0.6, *p* = 0.2193); ^b^ Random effects included in the model were block (df = 1, χ^2^ = 34.0, *p* < 0.0001), block × hybrid (df = 1, χ^2^ = 3.9, *p* = 0.0241), block × strain (df = 1, χ^2^ = 3.0, *p* = 0.0416), and block × hybrid × strain (df = 1, χ^2^ = 2.2, *p* = 0.0690); ^c^ The random effect of block (df = 1, χ^2^ = 6.9, *p* = 0.0043) was included in the model.

**Table 2:** Goodness of fit, LC_50_, and fiducial limits for diet-based bioassays with Cry3Bb1.

Strain	df	χ^2^	*p*	LC_50_^a^ (95% FL)
Susceptible	3	1.57	0.6653	6.09 (2.22 to 10.01)
Heterozygote	3	2.93	0.4022	32.90 (19.53 to 49.74)
Monona	3	8.08	0.0443	>341.60

^a^ LC_50_ is the lethal concentration that kills 50% of the population, and was measured in µg Cry3Bb1/cm^2^.

**Table 3:** Analysis of variance for the fitness cost experiment with Monona.

Analysis	Effect	df	F	*p*
Development rate	Strain	1,58	0.03	0.8651
	Sex	1,58	11.14	0.0015
	Strain × Sex	1,58	0.03	0.8537
Survival	Strain	1,30	0.18	0.6713
Size	Strain	1,58	0.69	0.6913
	Sex	1,58	0.02	0.9018
	Strain × Sex	1,58	0.25	0.6186
Adult lifespan	Strain	1,58	0.52	0.4746
	Sex	1,58	3.02	0.0877
	Strain × Sex	1,58	0.70	0.4059
Egg viability	Strain	1,30	0.71	0.4072

**Table 4:** Repeated-measures analysis of variance for fecundity.

Experiment	Effect	df	F	*p*
Susceptible vs. Monona	Strain	1,30	0.62	0.4370
	Week	8,227	78.74	<0.0001
	Strain × Week	8,227	1.21	0.3720
Susceptible vs. Elma	Strain	1,37	0.03	0.8681
	FA ^a^	1,37	8.51	0.0060
	SCR ^b^	1,37	1.58	0.2164
	Strain × FA	1,37	1.40	0.2438
	Strain × SCR	1,37	0.70	0.4083
	FA × SCR	1,37	1.17	0.2863
	Strain × FA × SCR	1,37	0.07	0.7965
	Week	6,169	30.67	<0.0001
	Strain × Week	6,169	0.43	0.8577
	FA × Week	6,169	5.97	<0.0001
	SCR × Week	6,169	1.32	0.2505
	Strain × FA × Week	5,169	1.53	0.1839
	Strain × SCR × Week	6,169	1.10	0.3655
	FA × SCR × Week	5,169	0.63	0.6764
	Strain × FA × SCR × Week	5,169	0.15	0.9788

^a^ FA = food availability, which was achieved by adding either more or fewer maize kernels to the larval rearing trays. See Methods for details; ^b^ SCR = interspecific larval competition through the presence or absence of southern corn rootworm larvae in larval rearing trays.

**Table 5:** Analysis of variance for the fitness cost experiment with Elma.

Analysis	Effect	df	F	*p*
Development Rate	Strain	1,78	7.73	0.0068
	FA ^a^	1,78	20.89	<0.0001
	Sex	1,78	15.31	0.0002
	SCR ^b^	1,78	1.02	0.3149
	Strain × FA	1,78	2.39	0.1260
	Strain × Sex	1,78	2.74	0.1017
	Strain × SCR	1,78	2.54	0.1149
	FA × Sex	1,78	7.59	0.0073
	FA × SCR	1,78	0.74	0.3938
	Sex × SCR	1,78	3.26	0.0749
	Strain × FA × Sex	1,78	0.49	0.4861
	Strain × FA × SCR	1,78	0.22	0.6434
	Strain × Sex × SCR	1,78	0.25	0.6175
	FA × Sex × SCR	1,78	0.00	0.9532
	Strain × FA × Sex × SCR	1,78	1.92	0.1695
Survival	Strain	1,50	0.54	0.4644
	FA	1,50	124.58	<0.0001
	SCR	1,50	34.2	<0.0001
	Strain × FA	1,50	0.02	0.8831
	Strain × SCR	1,50	0.08	0.7827
	FA × SCR	1,50	1.33	0.2540
	Strain × FA × SCR	1,50	0.21	0.6466
Size	Strain	1,73	0.04	0.8363
	FA	1,73	1.25	0.2668
	Sex	1,73	1.84	0.1792
	SCR	1,73	3.94	0.0508
	Strain × FA	1,73	0.08	0.7718
	Strain × Sex	1,73	0.00	0.9944
	Strain × SCR	1,73	0.41	0.5225
	FA × Sex	1,73	1.17	0.2830
	FA × SCR	1,73	0.03	0.8546
	Sex × SCR	1,73	0.97	0.3279
	Strain × FA × Sex	1,73	0.00	0.9649
	Strain × FA × SCR	1,73	0.00	0.9907
	Strain × Sex × SCR	1,73	0.33	0.5677
	FA × Sex × SCR	1,73	0.02	0.8918
	Strain × FA × Sex × SCR	1,73	0.33	0.5664
Adult Lifespan	Strain	1,74	0.32	0.5732
	FA	1,74	2.39	0.1261
	Sex	1,74	3.01	0.0871
	SCR	1,74	0.47	0.4952
	Strain × FA	1,74	0.1	0.7563
	Strain × Sex	1,74	0.04	0.8405
	Strain × SCR	1,74	0.04	0.8369
	FA × Sex	1,74	3.94	0.0509
	FA × SCR	1,74	1.24	0.2698
	Sex × SCR	1,74	1.47	0.2297
	Strain × FA × Sex	1,74	0.01	0.9417
	Strain × FA × SCR	1,74	0.36	0.5493
	Strain × Sex × SCR	1,74	0.68	0.4123
	FA × Sex × SCR	1,74	3.35	0.0712
	Strain × FA × Sex × SCR	1,74	0.63	0.4304
Egg Viability	Strain	1,18	0.27	0.6111
	FA	1,18	0.5	0.4891
	SCR	1,18	0.37	0.5486
	Strain × FA	1,18	0.26	0.6149
	Strain × SCR	1,18	0.16	0.6907
	FA × SCR	1,18	0.98	0.3349
	Strain × FA × SCR ^c^	-	-	-

^a^ FA = food availability, which was achieved by adding either more or fewer maize kernels to the larval rearing trays. See Methods for details; ^b^ SCR = interspecific larval competition through the presence or absence of southern corn rootworm larvae in larval rearing trays; ^c^ The interaction of strain × FA × SCR could not be calculated because cages with Elma from seedling mats with low food availability and SCR did not result in enough eggs to test egg viability.
